# A Universal Physics-Based Model Describing COVID-19 Dynamics in Europe

**DOI:** 10.3390/ijerph17186525

**Published:** 2020-09-08

**Authors:** Yiannis Contoyiannis, Stavros G. Stavrinides, Michael P. Hanias, Myron Kampitakis, Pericles Papadopoulos, Rodrigo Picos, Stelios M. Potirakis

**Affiliations:** 1 Department of Electrical and Electronics Engineering, University of West Attica, 12244 Athens, Greece; yiaconto@uniwa.gr (Y.C.); ppapadop@uniwa.gr (P.P.); spoti@uniwa.gr (S.M.P.); 2 School of Science and Technology, International Hellenic University, 57001 Thessaloniki, Greece; 3 Physics Department, International Hellenic University, 65404 Kavala, Greece; mhanias@physics.ihu.gr; 4 Major Network Installations Dept, Hellenic Electricity Distribution Network Operator SA, 18547 Athens, Greece; m.kampitakis@deddie.gr; 5 Physics Department, University of Balearic Islands, 07122 Palma Majorca, Spain; rodrigo.picos@uib.es

**Keywords:** COVID-19, model of the infection diffusion, self-organizing systems, lattice simulations, epidemiology, preventive measures

## Abstract

The self-organizing mechanism is a universal approach that is widely followed in nature. In this work, a novel self-organizing model describing diffusion over a lattice is introduced. Simulation results for the model’s active lattice sites demonstrate an evolution curve that is very close to those describing the evolution of infected European populations by COVID-19. The model was further examined against real data regarding the COVID-19 epidemic for seven European countries (with a total population of 290 million) during the periods in which social distancing measures were imposed, namely Italy and Spain, which had an enormous spread of the disease; the successful case of Greece; and four central European countries: France, Belgium, Germany and the Netherlands. The value of the proposed model lies in its simplicity and in the fact that it is based on a universal natural mechanism, which through the presentation of an equivalent dynamical system apparently documents and provides a better understanding of the dynamical process behind viral epidemic spreads in general—even pandemics, such as in the case of COVID-19—further allowing us to come closer to controlling such situations. Finally, this model allowed the study of dynamical characteristics such as the memory effect, through the autocorrelation function, in the studied epidemiological dynamical systems.

## 1. Introduction

Models on epidemic spread that take into account the self-organized criticality (SOC) of a virus [1,2] have been developed in the recent past [3,4,5]. In this direction, diffusion rules similar to the sandpile model of Bak-Tang-Wiesenfeld (BTW) [1] have been imposed, and the critical slowing-down (CSD) phenomenon has been thoroughly studied—among other fields, in epidemiology [6,7,8]. Within this framework, the aim of a self-organizing analysis of the coronavirus (COVID-19) pandemic globally using self-organizing maps has been accomplished [9]. Lately, besides self-organizing models, a variety of numerous other modeling (lattice model, network model, mathematical model, etc.) approaches regarding COVID-19 epidemics have been developed [10,11,12,13,14,15,16,17,18,19,20,21,22,23,24]; among these, the compartmental SIR (Susceptible, Infectious, or Recovered) models exhibit very interesting results, which are discussed below in Section 4.

The presented work is based on the fundamental notion of universality (universality classes), which is a basic concept in the physics of critical phenomena [25]. According to this, systems sharing no common structures and features at all may demonstrate the same dynamic behavior; in the case of critical phenomena, this is expressed through the existence of exponents in scaling laws. A characteristic example of universality is the phase transition from quark matter to hadron matter, which exhibits exactly the same dynamics as the critical state of Ising magnetic models [26]. Thus, the research motivation for the present work was to investigate whether a self-organizing diffusion over a lattice could describe viral epidemics, with a focus on the case of COVID-19 disease—although the described model appears to be a general approach for describing the spread of virus infection over a population.

In this paper, a general, self-organizing diffusion model (SODM) applied over a lattice is proposed. The proposed model is very simple in terms of the implementation of specific diffusion rules and incorporates only one control parameter. On each site of the lattice, a quantity called charge is placed, and we investigate how this diffuses from site to site under the specific rules set by the implemented SODM. In the general form of the model, this charge could be any physical quantity, such as energy (charge), electric charge, sandpile-grain charge, information charge, stock market charge, etc. Additionally, the model is designed with providence for the activity levels of the sites and communication restrictions among them.

In terms of the human society over which the epidemic is diffused, the investigated quantity is the dynamical (temporal) evolution of the infected population; on the model’s lattice, this is translated to the dynamical evolution of active sites (in algorithmic time units). This does not mean that viruses are spread over the lattice, avoiding a metaphor that is far from reality and in which concepts such as medical viral charge are involved, which have completely different properties from diffusion; for example, natural quantities such as energy, electric charge, etc. According to the above, the proposed approach cannot be included in any of the hitherto known categories of epidemiological models. It must be noted that, according to a work currently under preparation [27], self-organizing diffusion dynamics are indeed critical dynamics that are expressed through intermittency [28,29]; therefore, in this paper, we shall simply refer to self-organized diffusion without delving into the details of its dynamics (which is the subject of future work). The presented SODM focuses on the case of COVID-19 disease—although the described model appears to be a general approach for describing the spread of virus infection over a population. The study concentrates on the period in which restrictive physical contact measures were put in place. We describe the existence of equivalent quantities between the SODM and the epidemic phenomenon, which would result in the same qualitative and quantitative characteristics being present in both cases. An example of these quantities is the correspondence between the active sites in SOMD and the COVID-19 infected population in the real world. In this case, the presented results show excellent qualitative agreement between the proposed SODM and real-world data, while by adapting (fitting) the model to the epidemiological data from seven European countries with a total population of 290 million, excellent quantitative agreement emerges as well. These results further suggest that the self-organizing diffusion model (SODM) may be the simplest equivalent dynamical model that underpins the underlying mechanism for describing epidemics, and it appears to hold as a universal approach for viral epidemics. Finally, this approach is further supported by the calculation of the autocorrelation function for the paradigms of the seven countries studied, which was possible due to the existence of the simulating model (since real-world data do not provide the necessary number of points for calculations). Interesting results regarding the memory of the studied epidemic systems emerge.

## 2. Materials and Methods

### 2.1. The Proposed Self-Organizing Diffusion Model (SODM)

By attempting to introduce a self-organizing diffusion model (SODM), we consider a lattice where a quantity named total charge (*Q*) is distributed over the lattice sites and expressed in arbitrary quantities called units of charge. Although the simplest case of a lattice—that of a square of side *L*—is initially utilized, the model is engineered in such a way that other geometries could be considered as well. The SODM is a very simple model based on two different kinds of nodes: active and inactive. The active nodes are those that can transfer charge to or receive it from their neighbors, whilst inactive nodes can only receive charge. Based on these, two fundamental quantities—the control parameter and the order parameter—are defined as follows:

- The control parameter is defined as the density $\rho =\frac{Q}{P}$, where *Q* is the total charge distributed over the whole lattice, expressed in charge units (*Q* = Σ*q*_ij_), and P is the total population; i.e., the number of sites of the (*L × L*) lattice, with *L* being the length of the lattice.
- The percentage of the active sites *S* over the lattice population *P* defines the order parameter $M=\frac{S}{P}$. The sites corresponding to those carriers (*M*) are named active sites. As a result, by following the evolution of *M*, a good quantitative description about the evolution of the diffusion can be obtained.

In the proposed model, the nearest neighbor approach is utilized, and the activity level is defined using the number *n* = 1, 2, 3, 4. According to this, the charge (*q*_ij_) diffusion from an active site takes place only for the four (since it is a square lattice) nearest neighbors in steps of *n*/4 regardless of the charge *q*_ij_ (in charge units) of the site, thus defining four levels of diffusion. Notice that this procedure ensures that the remaining charge is always greater than or equal to zero. For instance, for *n* = 1 (level 1), each site may pass one-quarter of a charge unit (i.e., 0.25) to each of its four nearest neighbors, while the active sites are those for which *q*_ij_ ≥ *n* = 1; correspondingly for *n* = 3, as active sites are considered for those with *q*_ij_ ≥ *n* = 3, all sites with a charge of *q*_ij_ = 0, 1, 2 are inactive sites. Thus, *n* defines the active sites, which are those with a charge of *q*_ij_ ≥ *n* in charge units, and the amount of the charge transferred from active sites to their neighbors is *q*_ij_ = *n*/4 charge units. Additional rules conditioning the model’s behavior are as follows:

- As mentioned above, for an *n*-level diffusion, each site can dispense its charge (*n*/4) to each of its nearest neighbors, regardless of how many charge units it owns (*q*_ij_). This charge transfer is controlled by a random process; thus, the process occurs with a probability of 0.5.
- This version of the model is closed; thus, it is not externally supplied with charge at all. This does not exclude the possibility of being considered as an open system after the proper modification to the rules concerning its perimeter points.
- Regarding the initial conditions, these have significant roles in the model, and they are distinguished into two categories:
  ○ In the first category, the available charge is initially distributed over the lattice sites in a random, uniform way [30]. As a result, some sites will initially be found with zero charge, while others will present a large amount of charge. Then, the initially distributed charge will be diffused over the whole lattice.
  ○ In the second category, the initially available charge is again randomly distributed, but this time only at the lattice boundary-sites. In this case, the charge is expected to diffuse from the perimeter towards the interior of the lattice.

With the aim of modeling a viral epidemic, which usually begins with incoming incidents from abroad, we opted for the latter case; thus, the diffusion from the perimeter to the center of the lattice was considered.

- Sweeping all lattice sites is a recurrent procedure. The order parameter—i.e., the percentage of the active carriers—as a function of the algorithmic time is registered (each unit of the algorithmic time is considered to be one lattice sweep). Thus, a time series corresponding to the temporal evolution of the percentage of the active sites is generated and comprises the simulation’s output.
- Isolation between all lattice sites can be implemented in a four-level communication approach, meaning that the capability to pass charge (communications) from one lattice site to its nearest neighbors (comprising its environment) can be reduced. In this model version (a square lattice), the possible contact reduction levels are as follows:
  ○ 75% reduction; i.e., each site transfers charge to 1 out of 4 of its nearest neighbors.
  ○ 50% reduction; i.e., each site transfers charge to 2 out of 4 of its nearest neighbors.
  ○ 25% reduction; i.e., each site transfers charge to 3 out of 4 of its nearest neighbors.
  ○ No reduction—in this case, charge may be transferred to all four nearest neighbors.

Finally, it is noted that, since the system is self-organized and its temporal evolution obeys the above-described rule set, our intervention is limited to the initial definition of the order parameter; i.e., the initial density *ρ*.

**Figure 1:**
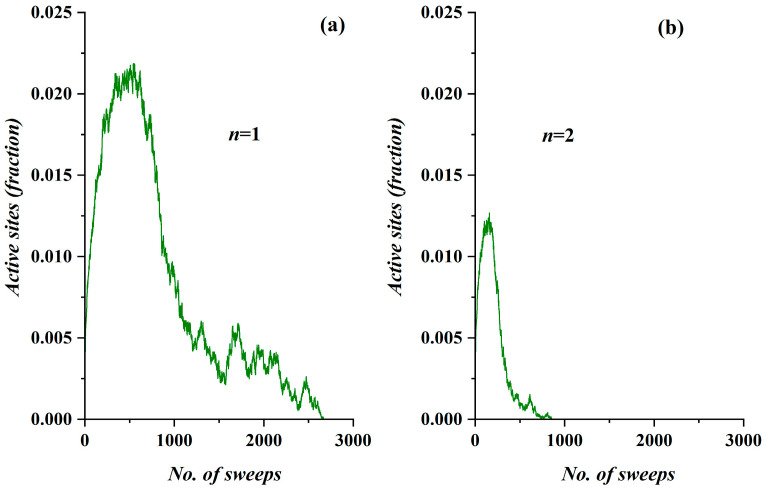
(**a**) The temporal evolution of the fraction of the active sites (order parameter) vs. the algorithmic time for the case *n* = 1, and (**b**) for the case *n* = 2. In both cases, the runs regarded a lattice with *L* = 250 sides, with communication capability restricted by 50% (2 out of 4 nearest neighbors may receive charge), and the same initial density of *ρ* = 0.055.

As an example of the proposed SODM system, the results generated in the case of a lattice with *L* = 250 sites and activity levels of *n* = 1 and *n* = 2, with the initial density set to *ρ* = 0.055, are shown in Figure 1. It is apparent that the diffusion is much stronger both in terms of intensity (i.e., the peak percentage of active sites) and duration in the case in which the activity level is set to *n* = 1 compared to *n* = 2. Additionally, the form of both plots appears to possess some general characteristics in common; specifically, the (algorithmically) temporal evolution of the order parameter (i.e., the fraction of the active sites *M*) clearly demonstrates three distinct phases: a steeper increase, a smoother relaxation and a kind of a narrow plateau. Regarding the role of the model’s lattice, it is noted that, although the utilized lattice was a square of 250 × 250 in size, the same results could be reproduced for any square lattice size, but for different values of the control parameter (initial density *ρ*). This feature does not lessen the value of the model; on the contrary, it further suggests its universality.

### 2.2. SODM and the COVID-19 Viral Epidemic

Considering the universal characteristics demonstrated by the SODM in the evolution curve, as shown in Figure 1, a comparison with the real data of the spread of the COVID-19 epidemic in Wuhan, China (see Figure 8 in [31]) revealed a significant similarity. This similarity motivated us to check the real epidemiological data of other countries; seven of them (all in Europe) are presented and discussed in this paper.

In advance of the presentation of the relevant results, the correspondence between the SODM parameters and the terms regarding epidemics is listed below:

- The active lattice sites of the SODM correspond to the infected active population due to an epidemic.
- The algorithmic time is measured in time units. Each time unit corresponds to a lattice sweep. It is apparent that the epidemic real time in days matches a number of lattice sweeps; i.e., a number of algorithmic time units.
- The charge in each site (*q*_ij_) in the SODM equivalently corresponds to the infection charge transferred during the spread of an epidemic and not to the virus charge in the medical sense (in the human body).
- The SODM models the infection diffusion and not the viral (in the medical sense) spread.
- The model incorporates four activity levels regarding the capability of a site to pass charge to its nearest neighbors. The strongest site activity (*n* = 1) means that even the site with the least charge (*q*_ij_ = 1 charge unit) is capable of diffusing its charge. For an epidemic, this means that even the carrier with the least virus charge (which should not be confused with the virus charge in the medical sense) can transmit the virus and indeed does so. Such a behavior could describe the most aggressive and easily diffused viruses, such as COVID-19. Thus, within the frame of the SODM, an activity level of *n* = 1 could describe epidemics caused by aggressive viruses. The diffusion of various, less aggressive, seasonal viruses, according to their characteristics, could be described by the other levels of activity with *n* > 1; for instance, for a less aggressive virus corresponding to activity level *n* = 2, the sites that have a charge *q*_ij_ = 1 charge unit are inactive, and so on.
- The effect of measures restricting physical contact (physical or social distancing) is also incorporated within the proposed SODM. As already described above, this is modeled in four levels due to the form of the lattice, which is a square. As a result, the allowed communication of a site with 1 out of its 4 nearest neighbors corresponds to restrictive measures of 75% (strict isolation policies), while less stringent distancing measures could be considered within the proposed SODM.

## 3. Results

In this section, the results of the model are fitted and compared to real epidemiological data from seven European countries. Additionally, thanks to the model’s accuracy, an initial study of the memory that the infection diffusion systems demonstrate, in the case of four European countries, is presented.

### 3.1. Comparative Results of the SODM and Real COVID-19 Epidemic Data

In this section, the relevant results regarding the application of the proposed novel self-organizing diffusion model (SODM) as compared to real epidemic data regarding the COVID-19 epidemic are presented. The real data regarded seven European countries with a total population of 290 million. These included four central European countries—namely France, Belgium, Germany and the Netherlands—that represent a population of more than 200 million in Europe, as well as Italy and Spain, which faced a severe (in terms of deaths and number of incidents) and extended spread, and Greece, which appears to be an example of timely, properly and successfully applied physical contact restriction measures. The data utilized were derived from the data base “Our World in Data” [32] and had the form of cumulative infection and death data for countries around the world.

**Figure 2:**
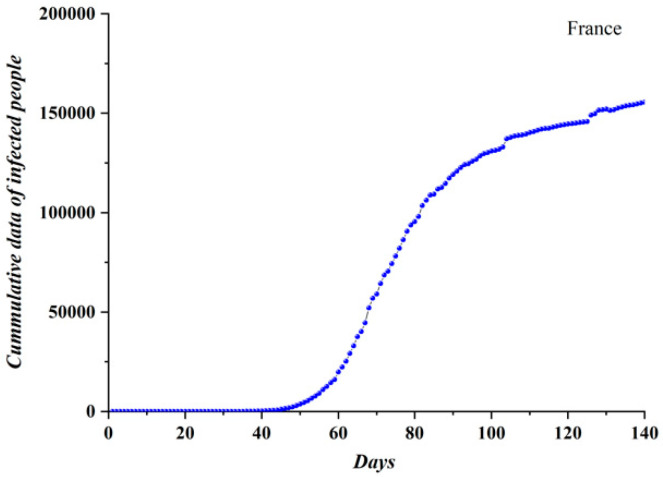
The cumulative COVID-19 infected population in France, as appears in [22]. The plotted period is from 18 February to 28 June 2020.

In Figure 2, the cumulative graph of the registered COVID-19-infected population in France appears. The presented graph illustrates the temporal evolution of the total number of registered infected people from 18 February 2020 until 26 June 2020; this period was chosen because during the first days of the epidemic, the fraction (percentage) of the infected people over the overall population was almost zero, while during the last days—within July—the restriction measures had almost been lifted.

**Figure 3:**
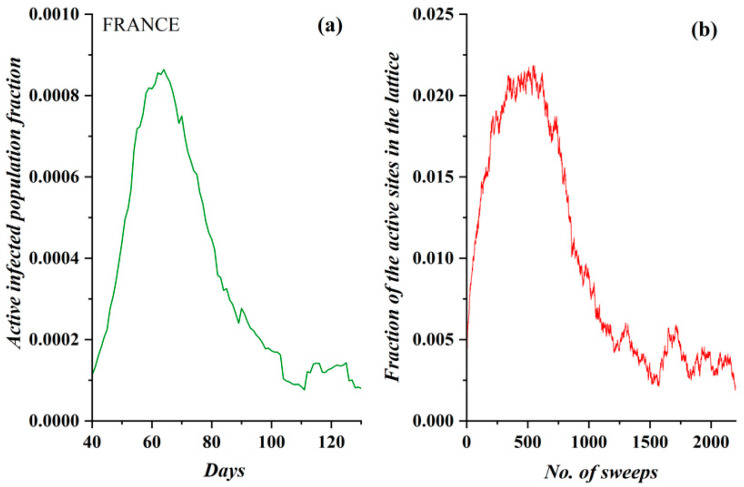
(**a**) The fraction of the active COVID-19 infected population daily in France (after 5 March 2020). Note that three phases are clearly distinguished. (**b**) The order parameter of the self-organizing diffusion model (SODM), namely the fraction of active lattice sites; the simulation vs. the number of lattice sweeps is presented for *ρ* = 0.055.

Since the SODM presents the temporal evolution of the fraction of the active sites (corresponding to the currently infected population), the available data had to be pre-processed accordingly so that they were comparable to the model’s results. To this end, pre-processing including the conversion of the cumulative infection data to data including the currently infected population daily was essential. In principle, this pre-processing excluded the dead and recovered people from the number of total incidents, as epidemiological studies have concluded that the average patient recovers within a maximum of 14 days [22,23]. Thus, the number of active (in diffusing the virus) infected people was estimated and is presented in Figure 3a for the case of France. Although the estimation of the infected population is therefore simple and safe, the first 14 days were removed from the data (and the graph); provided that, during the first days of the epidemic, there were few cases, the loss of information is negligible. The presented, pre-processed epidemiological data for France demonstrate the same characteristics that the SODM model possesses; i.e., there are three distinct phases, with a steep rise, a short plateau and a smooth relaxation (with reference to Figure 2).

Considering the fact that there is a qualitative similarity between the simulation results presented in Figure 3 (*n* = 1, *L* = 250, *ρ* = 0.055 and restriction measures of 50%) and the real data regarding COVID-19 diffusion in France during the period in which this country implemented certain restrictive measures, we initially deduced that the activity level of *n* = 1 properly described the diffusion behavior of an aggressive virus such as COVID-19. For the sake of clarity, the diffusion’s temporal evolution according the SODM in this case also appears in Figure 3b.

**Figure 4:**
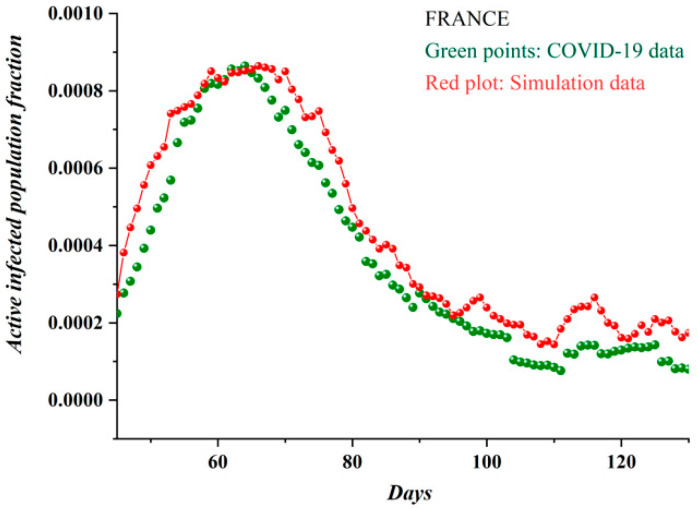
The COVID-19 epidemic data for France in green points, fitted by the proposed self-organized diffusion model (SODM) simulation data, in red points. Each day corresponds to 24 sweeps.

In order to extend the apparent qualitative similarity between Figure 3a,b—i.e., real epidemiological data—and the SODM to a quantitative confirmation, a fitting process was followed. According to this process, an axes normalization of the model’s plot was applied. For the horizontal axis, this was achieved by mapping the 90 days of real data (appearing in Figure 3a) to the 2200 sweeps (algorithmic time units, appearing in Figure 3b), thus resulting in a value of 24 lattice sweeps per day. To create the fitting, for every 24 lattice sweeps, the mean value of the simulated data presented in Figure 3b was calculated. The new SODM time series was extended with the same time scale as the real data. Normalization on the vertical axis was simply achieved by fitting the scaling of the model to the scaling of the real data; i.e., the simulation points were divided by a factor arising from the ratio of the model’s peak value to the real data peak value, and the proper offset (when necessary) was added. The resulting fitting of the model to real data is shown in Figure 4; the COVID-19 epidemic data in France (green points) are fitted by the proposed SODM simulation data (red points and plot). It is apparent from this graph that there is an impressive agreement between the real data and the model.

As mentioned above, in an attempt to check the validity of our model, we have repeated the described procedure of data pre-processing and SODM model fitting for six more European countries: Germany, Belgium, the Netherlands, Spain, Italy and Greece. It is noted that all the countries imposed social distancing (restrictions of physical contact), with Italy not implementing them in a timely manner, and that Belgium and Greece have almost the same population. Since the proposed SODM is a very simple model, the definition of the proper simulation set of parameters included the initial charge density *ρ* and the level of social distancing measures imposed. Consequently, it was necessary to investigate the initial charge density *ρ* on the boundaries of the lattice and the level of restricting measures, which ranged from the case of no restrictive measures to 25% (transfer charge to 3 out of 4 nearest neighbors), 50% (transfer charge to 2 out of 4 nearest neighbors) and 75% (transfer charge from 1 to 4 nearest neighbors) limitations on physical contact.

**Figure 5:**
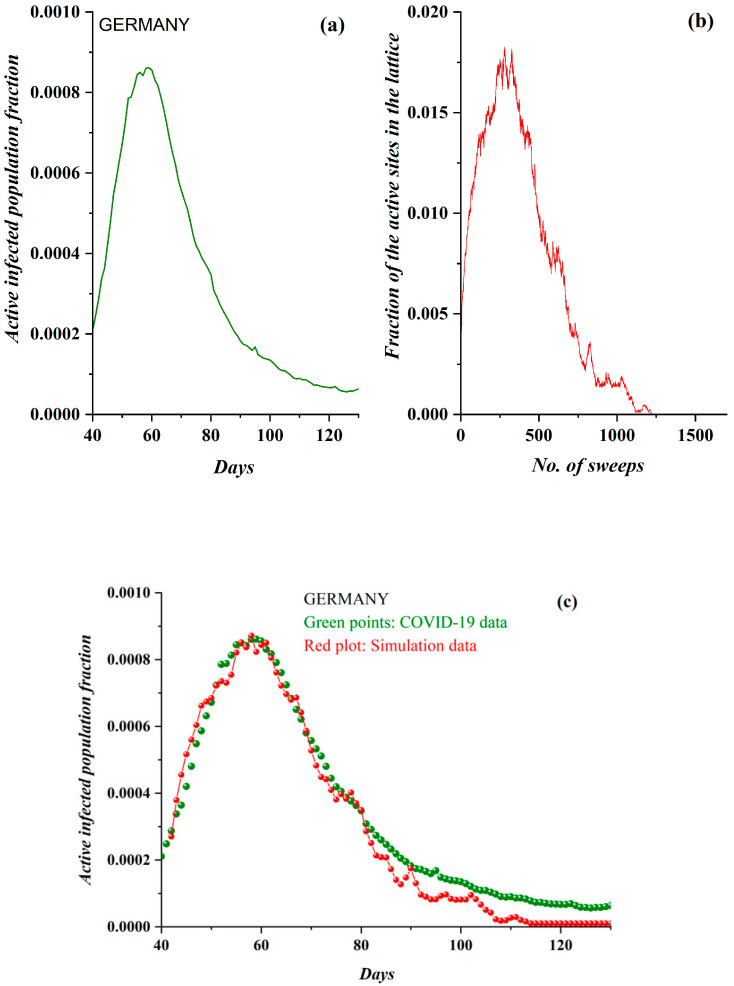
(**a**) The fraction of the active COVID-19-infected population daily in Germany (after 5 March 2020). Again, the three phases are clearly distinguished. (**b**) The fraction of active lattice sites (order parameter of the SODM) vs. the number of lattice sweeps is shown. (**c**) The COVID-19 epidemic data for Germany in green points, fitted by the proposed a self-organized diffusion model (SODM) simulation data, in red points. Each day corresponds to 22 sweeps.

In Figure 5a, the pre-processed COVID-19 data in the case of Germany and the derived SODM simulation results (Figure 5b) are shown. They demonstrate an impressive qualitative similarity. The data regarded the period after 3 March 2020, while simulation results were produced for an initial charge density of *ρ* = 0.040 and a level of restrictive measures of 50% (as, on average, no total lockdown was implemented in Germany). In Figure 5c, the model’s fitting on the real data, according to the previously described methodology (each day corresponds to 22 sweeps), reveals an even more impressive agreement.

**Figure 6:**
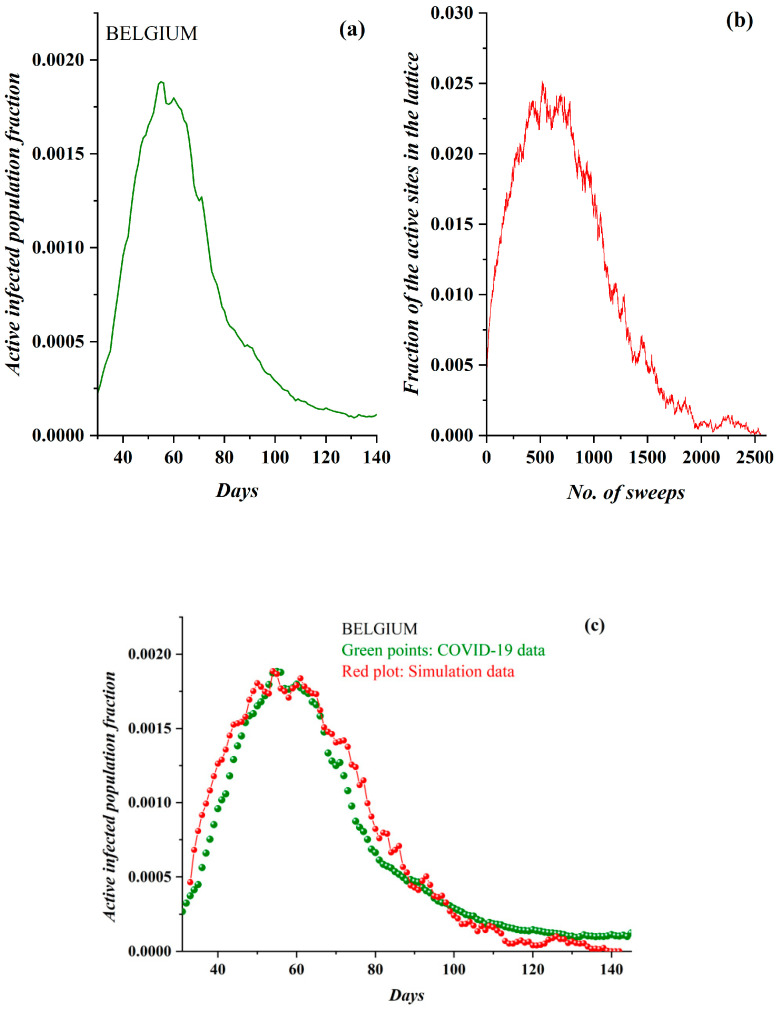
(**a**) The fraction of the active COVID-19-infected population daily in Belgium (after 1 March 2020). The three phases are clearly distinguished. (**b**) The fraction of active lattice sites (order parameter of the SODM) vs. the number of lattice sweeps is shown. (**c**) The COVID-19 epidemic data for Belgium in green points, fitted by the proposed a self-organized diffusion model (SODM) simulation data, in red points. Each day corresponds to 23 sweeps.

Correspondingly, in Figure 6a, the pre-processed COVID-19 data in the case of Belgium and the derived SODM simulation results (Figure 6b) are shown. They also demonstrate a full qualitative similarity. The data regarded the period after 1 March 2020, while simulation results were produced with an initial charge density of *ρ* = 0.059 and a level of restrictive measures of 50% (as, on average, no total lockdown was implemented). In Figure 6c, the model’s fitting to the real data, according to the previously described methodology (this time each day corresponds to 23 sweeps), also reveals an impressive agreement.

**Figure 7:**
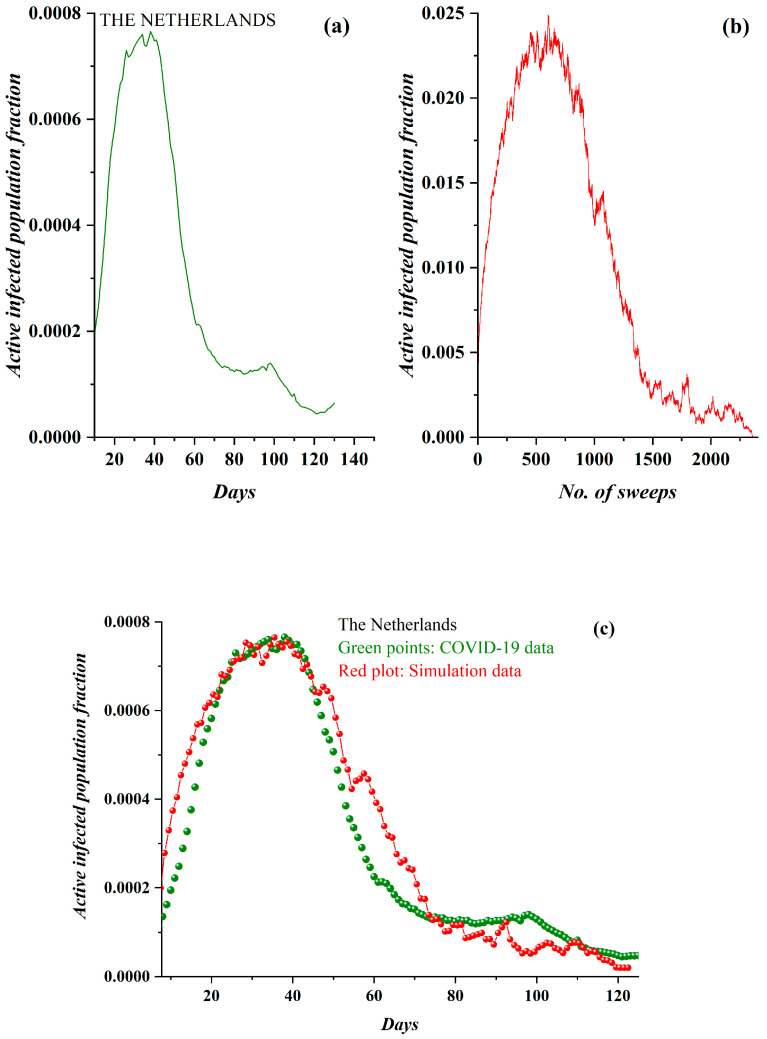
(**a**) The fraction of the daily active COVID-19-infected Dutch population (after 10 March 2020). The three described phases are clearly distinguished. (**b**) The fraction of active lattice sites (order parameter of the SODM) vs. the number of lattice sweeps is shown. (**c**) The COVID-19 epidemic data for the Netherlands in green points, fitted by the proposed SODM simulation data, in red points. Each day corresponds to 21 sweeps.

Likewise, in Figure 7a, the pre-processed COVID-19 data in the case of the Netherlands and the derived SODM simulation results (Figure 7b) are presented. Again, full qualitative similarity between the model and real data can be observed. The data regarded the period after 10 March 2020, while simulation results were produced with an initial charge density of *ρ* = 0.057. As, on average, no total lockdown was implemented in the Netherlands, the level of restrictive measures was set to 50%. In Figure 7c, the model’s fitting to the real data according to the described methodology (each day corresponds to 21 sweeps) again reveals a very good agreement.

**Figure 8:**
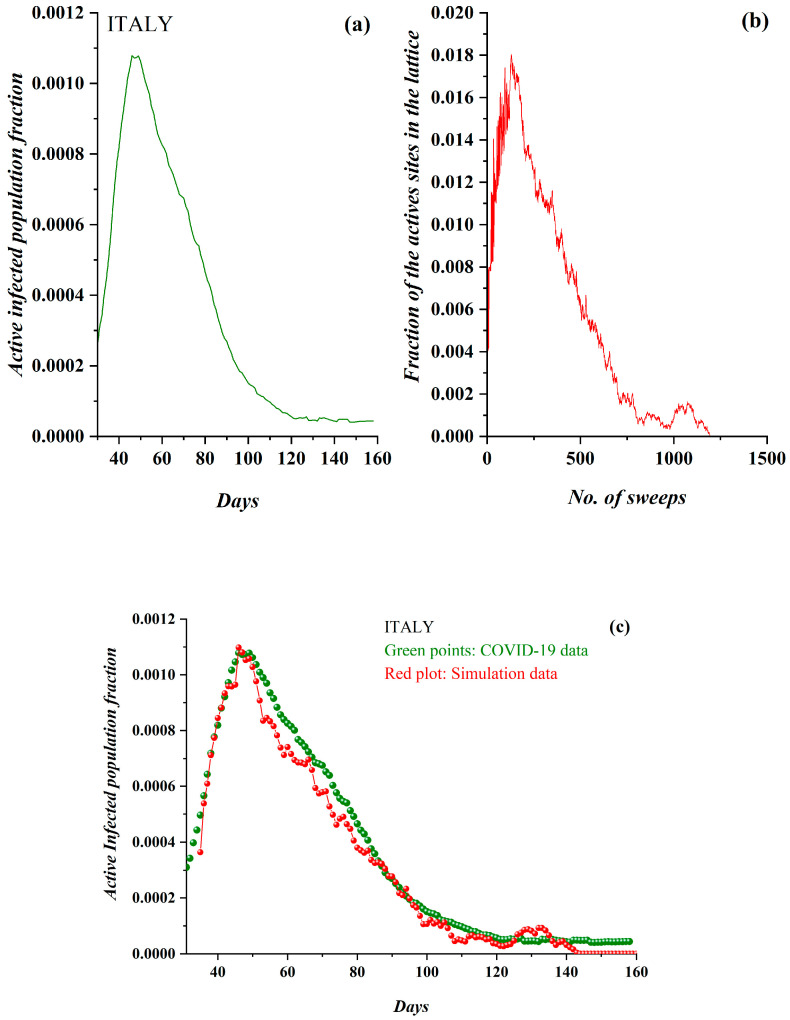
(**a**) The fraction of the active COVID-19-infected population daily in Italy (from 1 March 2020). (**b**) The fraction of active lattice sites in the SODM simulation vs. the number of lattice sweeps, is shown. (**c**) The epidemiological data for Italy in green points, fitted by the SODM simulation data, in red points. Note that each day corresponds to 11 sweeps.

The next country studied was the first to face a COVID-19 epidemic in Europe. Italy was struck in February 2020 and did not impose social distancing measures in a timely manner; on the contrary, these were imposed in a later stage of the epidemic spread and did not include an immediate total lockdown. For this reason, the attempted SODM simulation did not include any restrictive measures of physical contact for the first 120 sweeps, while for the rest of the studied period, these were set to 50%. The initial density, providing a good fitting of the model to real data, was *ρ* = 0.386. In Figure 8a, the pre-processed COVID-19 data and the derived SODM simulation results (Figure 8b) are presented, demonstrating full qualitative similarity between the model and real data. The epidemiological data regarded the period after 1 March 2020. Although the real data in the case of Italy included a rather acute peak, the SODM qualitatively simulated the evolution successfully. In Figure 8c, the model’s fitting to the real data according to the described methodology (each day corresponds to 11 sweeps) shows excellent agreement.

**Figure 9:**
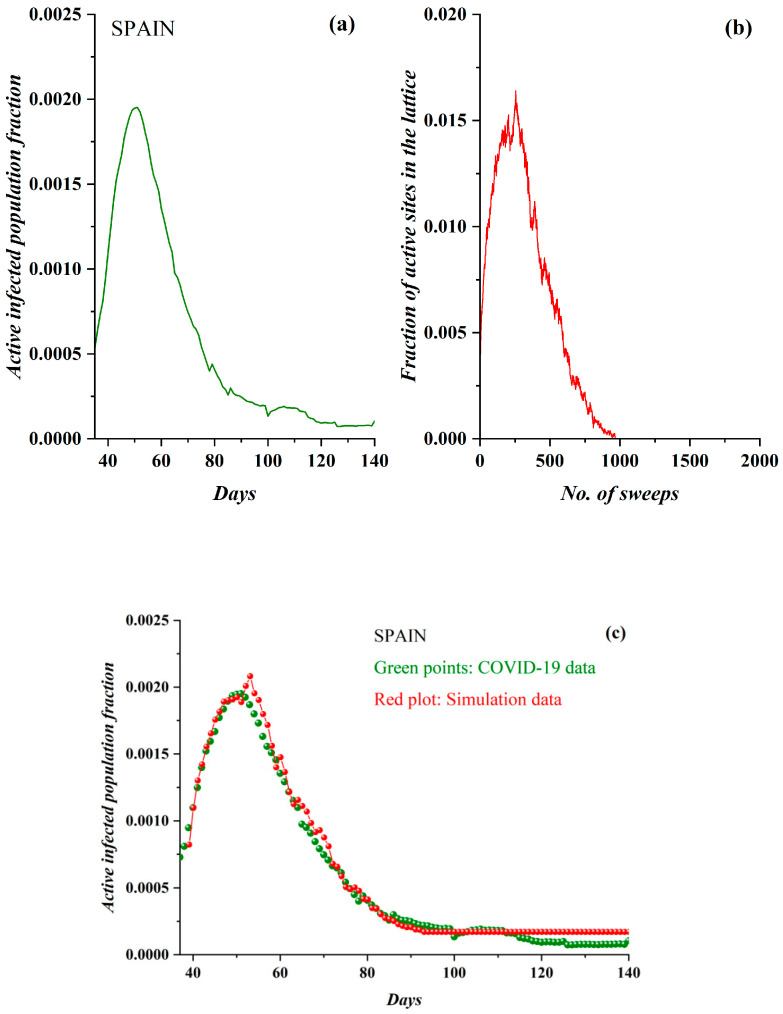
(**a**) The fraction of the active COVID-19-infected population daily in Spain (from 1 March 2020). (**b**) The fraction of active lattice sites; the simulation vs. the number of lattice sweeps is shown. (**c**) The COVID-19 epidemic data in green points, fitted by the proposed the SODM simulation data, in red points. In the case of Spain, each day corresponds to 18 sweeps.

Spain was the second European country (after Italy) to be struck by COVID-19, and it at first reacted slowly, allowing for an initial rapid and aggressive epidemic spread. Social distancing measures allowed for the spread to be slowed; the pre-processed data are shown in Figure 9a. The derived SODM simulation results appear in Figure 9b, for *ρ* = 0.0378 and restrictive measures of 50%; again, full qualitative similarity between the model and real data is demonstrated, although the real data again included a rather acute peak (compared to the rest of the cases). The presented epidemiological data regarded the period after 1 March 2020. In Figure 9c, the model’s fitting to the real data is shown, considering 18 sweeps to correspond to one day, clearly demonstrating excellent agreement with the real epidemiological data.

**Figure 10:**
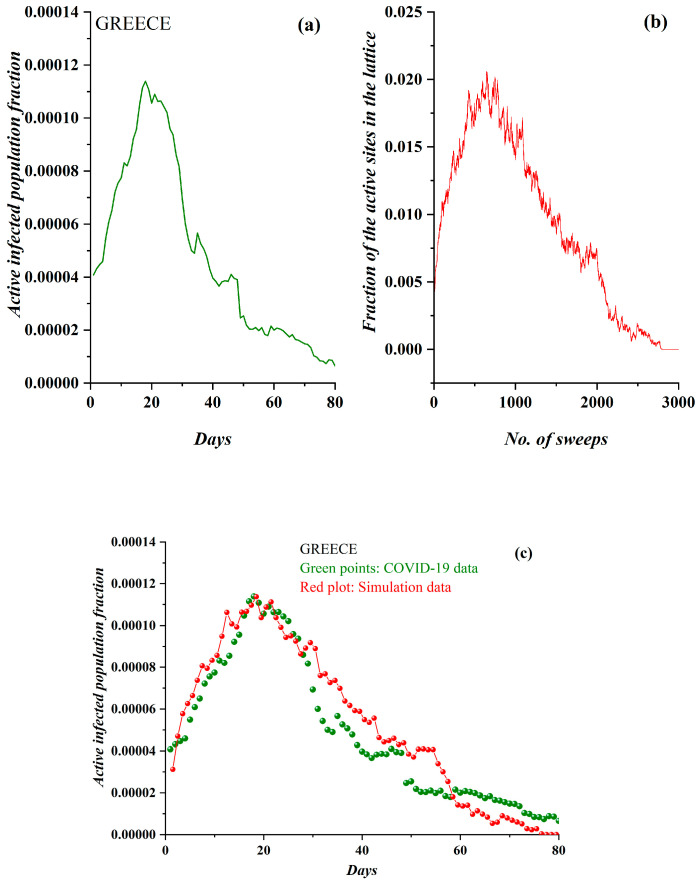
(**a**) The fraction of the active COVID-19-infected population daily in Greece (from 5 March 2020). (**b**) The order parameter of the SODM, namely the fraction of active lattice sites—simulation vs. the number of lattice sweeps—is shown. (**c**) The COVID-19 epidemic data for Greece in green points, fitted by the proposed a self-organized diffusion model (SODM) simulation data, in red points. Each day corresponds to 37 sweeps.

Finally, in Figure 10, the epidemiological data and the SODM simulation results appear in the case of Greece, for the period before 1 July 2020, when restrictive measures of all kinds ceased. Greece is a noteworthy case because it successfully faced the challenge set by the COVID-19 epidemic, demonstrating an extremely low number of incidents and deaths. Specifically, enhanced restrictive measures in the form of a total lockdown were implemented quickly. The result of such a policy is clearly depicted in Figure 10a, where the pre-processed COVID-19 data for Greece are shown. The derived SODM simulation results are presented in Figure 10b and they also demonstrate a qualitative similarity. The data regarded the period after 5 March 2020. In this case, simulation results were produced with an initial charge density of *ρ* = 0.0455 and a level of restrictive measures of 75% (the highest), since a total lockdown was imposed. Correspondingly, in Figure 10c, the model’s fitting to the real data according to the previously described methodology (where one day corresponds to 37 sweeps) also reveals a good agreement in this case.

### 3.2. Autocorrelation Function of Epidemic Spread

In an attempt to obtain further insights on the dynamic mechanism of epidemics, an investigation of the possible memory that the relevant dynamics demonstrate is useful. It is known that the autocorrelation function is connected to the memory of a system [33]. Thus, in the case of an epidemic, the autocorrelation function could provide an estimation of the memory retention of an epidemic’s temporal evolution. It is apparent that an epidemic with a strong memory renders a dangerous situation that could sustain the epidemic for a long time.

On the other hand, epidemiological data are daily records over a specific period, and thus they are not sufficient to calculate the corresponding autocorrelation function properly and reliably. If, instead of the real data, the data points produced by the SODM model were utilized, the corresponding autocorrelation function could be reliably calculated, under the restriction that the model properly fits the real data.

The above idea was implemented and is presented for seven European countries. As mentioned above, the simulation data for each country are presented in Figure 3b, Figure 5b, Figure 6b and Figure 7b, respectively. The autocorrelation function *C*(*m*) for a timeseries *x*_1_, *x*_2_, *x*_3_, ……, *x_n_* is defined by

(1) $$C\left(m\right)=\frac{1}{n-m}\overset{n-m}{\underset{i=1}{\sum }}(x_{i}-<x>)\left(x_{i+m}-<x>\right)$$

where *m < n* and

(2) $$<x>=\frac{1}{n}\overset{n}{\underset{i=1}{\sum }}x_{i}$$

**Figure 11:**
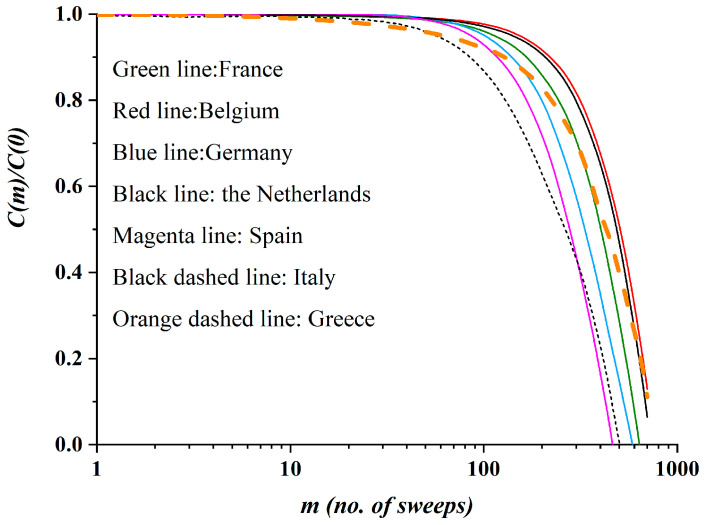
The normalized autocorrelation functions, as calculated by the proposed SODM simulation data; France is shown by the green line, Belgium by the red line, Germany by the blue line, the Netherlands by the black line, Spain by the green line, Italy by the black dashed line and Greece by the orange dashed line. A classification of the countries according to the extent of restrictive measures is apparent.

In Figure 11, the autocorrelation function for all four countries mentioned above appears on the same graph for comparison. The correlation was extended up to a maximum correlation length of *m*_max_ = 700 simulation data points. This is translated to a correlation time interval of about 30 days.

It is apparent from Figure 11 that, in all cases the autocorrelation function has almost the same form. This reinforces the universal character of the proposed SODM epidemiological model. Up to *m* = 100—i.e., for a time interval of almost the first five days—very strong temporal correlations are shown for all the countries, with the exception of Greece. This means that a strong memory has been developed by the dynamical system describing the corresponding epidemics during the rise of the phenomenon (the increase of the active, infected population). In the peak of each curve, a separation of the correlations for each case appears; that is, the peculiarities of each country emerge. After that, the correlations appear to fall, meaning that the memory of the dynamical systems weakens. It is noted that Belgium’s simulation data imply a system with a higher memory than that for the rest of the countries, which implies a more dangerous situation for the epidemic in this country.

Considering the same figure (Figure 11), an interesting phenomenon appears. In the cases of the six countries—namely France, Belgium, Germany, the Netherlands, Spain and Italy—the autocorrelation function graph has almost the same form; actually, all six graphs belong to the same family of curves. It is noted that these six countries belong to the same category of restrictive measures (an average of 50% reduction in physical contacts, for the studied period). However, in the case of Greece, which adopted restrictive measures corresponding to an average 75% reduction in physical contacts (i.e., a total lockdown for almost all of the studied period), the autocorrelation function graph does not belong to the family of curves of the other six countries; furthermore, it clearly intersects all of them. These results clearly suggest a classification of the countries according to the extent of the imposed restrictive measures of physical contact. Thus, we expect a categorization of the autocorrelation diagrams whereby countries with the same restrictive measures belong to the same family of curves. One explanation could be that the amplitude of the fluctuations in the values of *M(t)* is different depending on the restrictive measures; stricter measures lead to suppressed fluctuations of the order parameter, and this is depicted in the autocorrelation function.

**Figure 12:**
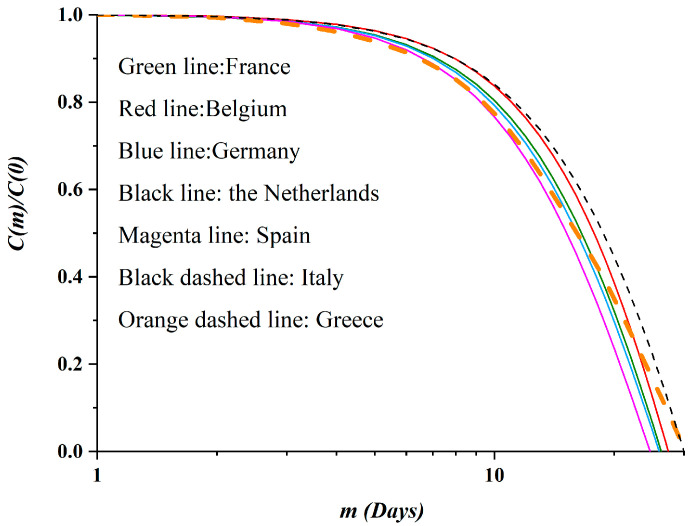
The normalized autocorrelation functions, as calculated by the epidemiological data; France is shown by the green line, Belgium by the red line, Germany by the blue line, the Netherlands by the black line, Spain with the green line, Italy by the black dashed line and Greece by the orange dashed line. Note that the plot corresponding to Greece intersects all the other plots, as in Figure 11.

Finally, although the number of points of the real data is not adequate to calculate the autocorrelation function directly, for the sake of comparison, we opted to additionally calculate it and present the results for all seven countries. The corresponding graphs appear in Figure 12. The behavior of the autocorrelation function appears to be qualitatively the same as those calculated by the SODM. The classification of the countries according to the level of restrictive measures is again evident, while Greece again intersects the graphs of the other six countries. Apparently, these results further support the validity of the proposed SODM; besides this, they support the possible emergence of a tool for classifying the evolution of epidemics in population sets according to the social distancing measures imposed.

## 4. Discussion

In this paper, a novel self-organizing diffusion model (SODM) is introduced. It is based on the approach of self-organizing systems—a mechanism widely used in nature, possessing the property of universality (i.e., the characteristic of directly or equivalently describing various systems), demonstrating at the same time a noteworthy simplicity. The proposed model is considered to be a closed one and has only a control and an order parameter, which are the charge *ρ* (in a general sense) and the fraction Μ of the currently active sites, respectively. The model is graded in the sense of both the activity and the allowed diffusion level. It describes a universal diffusion behavior under the conditions imposed by the self-organizing principle. The feature of universality emerges through the correlations developed within the systems.

However, the proposed model is capable of mapping an epidemic’s general features (i.e., various aggressiveness levels to activity levels) and their temporal evolution. Additionally, it incorporates the ability to include the influence of restrictive measures (through four-level communication) imposed by governing entities on sets of populations. Thus, lattice dynamics within the SODM are proposed to be an equivalent dynamical system that is able to describe the dynamics of epidemics in general. It is noted that, due to the mentioned universal character of the proposed SODM, no correspondence between the parameters utilized for describing the particularities of an epidemic/pandemic, as appear in the relevant epidemics or medicine terminology (such as R0, etc.), should be expected.

The simulation results of the introduced SODM—in the version of modeling the most aggressive case (activity level *n* = 1) with restrictive measures imposed—reveal that the spread of COVID-19 over a population obeys this self-organizing diffusion process, which is a simple and preferred approach in nature. Motivated by this similarity and the fact that most of the real data regarding the COVID-19 epidemic in various countries qualitatively demonstrate the same curve for the temporal evolution of the fraction of the active infected people (*M*), an investigation of the direction describing the evolution of COVID-19 epidemics in European countries was attempted. By adjusting the appropriate parameters (i.e., the initial density *ρ* and the average communication level) in the SODM, the simulations allow for the approximation/fitting of the evolution of the epidemics in the studied cases. Thus, a better understanding of the relevant underlying dynamics is achieved.

The presented results show an extremely good fitting of simulation results to the epidemiological data (quantitative agreement) in the cases of the seven studied European countries. The studied countries demonstrated different epidemic evolutions due to different approaches or policies in facing COVID-19 and could be called representative cases. These results regarded countries that finally imposed restrictions on physical contact at various levels and scientifically document the view that they are indeed effective in controlling epidemics. This is clearly proven by comparing the case of Belgium (a medium scale of restrictions and not implemented in a timely manner) to that of Greece (strict restriction measures, reducing physical contact by almost 80%). Although they practically have the same population, both the peak and the duration of the epidemic were significantly reduced in the case of Greece (by an order of magnitude). Since during these difficult times of the COVID-19 pandemic, controversy over the proper approach in managing this situation is present worldwide, the model offers a scientific documentation of the effectiveness of restrictions, beyond empirical studies.

Another issue investigated in this paper that enhances our understanding of the dynamics demonstrated by epidemics was the possible memory effect exhibited. An epidemic with a strong memory is a dangerous situation that could sustain the epidemic for a long time. This was investigated by calculating the autocorrelation function for all seven European countries (France, Germany, Belgium, the Netherlands, Italy, Spain and Greece). The first six countries demonstrated almost the same epidemiological characteristics. This was made possible by utilizing the results of the SODM, since real data points are not sufficient for such evaluations. It was found that all countries initially exhibited a strong memory during the rise of the epidemic, with Greece being less persistent. In the peak of the epidemic curve of each country, a separation of the correlations for each case appeared; that is, the peculiarities of each country emerged. During the relaxation of the epidemic, the memory effect weakened. An interesting observation is the fact that the autocorrelation function appears to offer a tool for classifying the level of restrictive measures on physical contact imposed by governing entities over sets of populations. It is demonstrated that countries with the same level of restrictive measures belong to the same family of curves regarding their autocorrelation graphs, further leading to the conclusion that the form of the epidemic’s relaxation depends on the restrictive measures imposed.

Comparing the results of the proposed SODM to the results produced by other approaches and methods, it is noted that these are closer to the SIR (susceptible–infected–recovered) models [34,35,36,37,38,39]. Since the general form of the SODM results is very close to the SIR curves, it would be interesting to investigate the connection between the two models and specifically the connection to the differential equation describing the evolution of the infected population (I). A good turning point could be to treat this differential equation as a Langevin equation. It is known that the Langevin equation can describe the order parameter of a second-order transition in critical phenomena [40]. In our model, the order parameter *M(t)* is the fraction of the active sites, corresponding to the infected population (*I*) of the SIR model, further providing a justification framework that is capable of explaining the similarities between the SODM and SIR models’ similarities.

Since the pattern describing the evolution of an epidemic appears to be a generalized one, based on a physical mechanism, future work should focus on an extensive and exhaustive number of simulations, including more countries, so that we can safely reach general conclusions on the dynamics of epidemics and possible classifications of the existing behaviors, according to the imposed measure-profiles of the studied countries. Such a work will contribute to more effectively handling the dynamics of epidemics and further allow a better understanding of them.

## 5. Conclusions

It seems that self-organization—a natural mechanism that is also applied in artificial (social, economic etc.) networks, due to its universal characteristics—appears to be applied in the case of virus-caused epidemics as well. The proposed novel self-organizing diffusion model (SODM)—describing a universal diffusion behavior in the simplest way—was shown to successfully fit the COVID-19 epidemics in seven European countries—namely France, Belgium, Germany, the Netherlands, Italy, Spain and Greece—while it seems that it has the potential to become a generalized model, equivalently describing the dynamics of epidemics and their features. This is claimed because almost all the real epidemiological data curves appear to follow the same general form. Since the model is structurally engineered to include restrictive contact rules, the value of restrictive measures is also documented by the model. Finally, the model allowed us to study dynamical characteristics, such as the memory effect, according to the relevant epidemiological data and provided us with a tool for quickly classifying countries according to the average restrictive measures imposed. Additionally, epidemic forecasting (with a focus on COVID-19) is another issue which we intend to study, including studying of lattice-forms that offer more choices in scaling social distancing measures, etc. The upcoming second wave of the COVID-19 pandemic will provide us with new epidemiological data that will ultimately test the limits of our approach. Finally, it should be mentioned that, according to ongoing research into self-organizing diffusion dynamics, various interesting results emerge in the frame of universality, the most important of which being the fact that self-organizing diffusion is governed by the dynamics of critical phenomena expressed through intermittency [29].

## References

[1] Bak P., Tang C., Wiesenfeld K. (1987). Self-organized criticality: An explanation of 1/f noise. Phys. Rev. Lett..

[2] Ion S., Marinoschi G. (2017). A self-organizing criticality mathematical model for contamination and epidemic spreading. Discret. Contin. Dyn. Syst. B.

[3] Rhodes C., Jensen H.J., Anderson R.M. (1997). On the critical behaviour of simple epidemics. Proc. R. Soc. B Biol. Sci..

[4] van de Leemput I.A., Wichers M., Cramer A.O.J., Borsboom D., Tuerlinckx F., Kuppens P., van Nes E.H., Viechtbauer W., Giltay E.J., Aggen S.H. (2014). Critical slowing down as early warning for the onset and termination of depression. Proc. Natl. Acad. Sci. USA.

[5] Stollenwerk N. (2005). Self-organized criticality in human epidemiology. AIP Conf. Proc..

[6] Brett T.S., Ajelli M., Liu Q.-H., Krauland M.G., Grefenstette J.J., Van Panhuis W.G., Vespignani A., Drake J.M., Rohani P. (2020). Detecting critical slowing down in high-dimensional epidemiological systems. PLoS Comput. Biol..

[7] Melin P., Monica J.C., Sanchez D., Castillo O. (2020). Analysis of Spatial Spread Relationships of Coronavirus (COVID-19) Pandemic in the World using Self Organizing Maps. Chaos Solitons Fractals.

[8] Nadim S.K.S., Ghosh I., Chattopadhyay J. (2020). Short-term predictions and prevention strategies for COVID-19: A model-based study. arXiv.

[9] Holmdahl I., Buckee C. (2020). Wrong but Useful—What Covid-19 Epidemiologic Models Can and Cannot Tell Us. N. Engl. J. Med..

[10] Weitz J.S., Beckett S.J., Coenen A.R., Demory D., Dominguez-Mirazo M., Dushoff J., Leung C.-Y., Li G., Măgălie A., Park S.W. (2020). Modeling shield immunity to reduce COVID-19 epidemic spread. Nat. Med..

[11] Chinazzi M., Davis J.T., Ajelli M., Gioannini C., Litvinova M., Merler S., Piontti A.P.Y., Mu K., Rossi L., Sun K. (2020). The effect of travel restrictions on the spread of the 2019 novel coronavirus (COVID-19) outbreak. Science.

[12] Zhao S., Chen H. (2020). Modeling the epidemic dynamics and control of COVID-19 outbreak in China. Quant. Biol..

[13] Giordano G., Blanchini F., Bruno R., Colaneri P., Di Filippo A., Di Matteo A., Colaneri M. (2020). Modelling the COVID-19 epidemic and implementation of population-wide interventions in Italy. Nat. Med..

[14] Ndaïrou F., Area I., Nieto J.J., Torres D.F. (2020). Mathematical modeling of COVID-19 transmission dynamics with a case study of Wuhan. Chaos Solitons Fractals.

[15] Gatto M., Bertuzzo E., Mari L., Miccoli S., Carraro L., Casagrandi R., Rinaldo A. (2020). Spread and dynamics of the COVID-19 epidemic in Italy: Effects of emergency containment measures. Proc. Natl. Acad. Sci. USA.

[16] Kucharski A.J., Russell T.W., Diamond C., Liu Y., Edmunds J., Funk S., Eggo R.M., Sun F., Jit M., Munday J.D. (2020). Early dynamics of transmission and control of COVID-19: A mathematical modelling study. Lancet Infect. Dis..

[17] Fanelli D., Piazza F. (2020). Analysis and forecast of COVID-19 spreading in China, Italy and France. Chaos Solitons Fractals.

[18] Bertozzi A.L., Franco E., Mohler G., Short M.B., Sledge D. (2020). The challenges of modeling and forecasting the spread of COVID-19. Proc. Natl. Acad. Sci. USA.

[19] Tsiotas D., Magafas L. (2020). The Effect of Anti-COVID-19 Policies on the Evolution of the Disease: A Complex Network Analysis of the Successful Case of Greece. Physics.

[20] Maugeri A., Barchitta M., Battiato S., Agodi A. (2020). Modeling the Novel Coronavirus (SARS-CoV-2) Outbreak in Sicily, Italy. Int. J. Environ. Res. Public Health.

[21] Demertzis K., Tsiotas D., Magafas L. (2020). Modeling and Forecasting the COVID-19 Temporal Spread in Greece: An Exploratory Approach based on Complex Network Defined Splines. Int. J. Environ. Res. Public Health.

[22] Rhodes C., Anderson R. (1997). Epidemic Thresholds and Vaccination in a Lattice Model of Disease Spread. Theor. Popul. Biol..

[23] Keeling M.J., Eames K.T.D. (2005). Νetworks and epidemic models. J. R. Soc. Interface.

[24] Kosmidis K., Macheras P. (2020). A fractal kinetics SI model can explain the dynamics of COVID-19 epidemics. PLoS ONE.

[25] Huang K., Holbrow C.H. (1965). Statistical Mechanics. Phys. Today.

[26] Wong C.-Y. (1994). Introduction to High-Energy Heavy-Ion Collisions.

[27] Contoyiannis Y., Stavrinides S.G., Hanias M.P., Kampitakis M., Papadopoulos P., Picos R., Potirakis S.M. (2020). Critical Lattice-Dynamics in Self-Organized Diffusion.

[28] Contoyiannis Y., Diakonos F. (2000). Criticality and intermittency in the order parameter space. Phys. Lett. A.

[29] Contoyiannis Y.F., Diakonos F.K., Malakis A. (2002). Intermittent Dynamics of Critical Fluctuations. Phys. Rev. Lett..

[30] Contoyiannis Y.F., Diakonos F.K. (2006). Abrupt transition in a sandpile model. Phys. Rev. E.

[31] Huang Y., Wu Y., Zhang W. (2020). Comprehensive identification and isolation policies have effectively suppressed the spread of COVID-19. Chaos Solitons Fractals.

[32] Our World in Data. https://ourworldindata.org/coronavirus-data-explorer?zoomToSelection=true&casesMetric=true&interval=total.

[33] Hassani H., Leonenko N., Patterson K. (2012). The sample autocorrelation function and the detection of long-memory processes. Phys. A Stat. Mech. Appl..

[34] Harko T., Lobo F.S.N., Mak M.K. (2014). Exact analytical solutions of the Susceptible-Infected-Recovered (SIR) epidemic model and of the SIR model with equal death and birth rates. Appl. Math. Comput..

[35] Beckley R., Weatherspoon C., Alexander M., Chandler M., Johnson A., Batt Ghan S. (2020). Modeling Epidemics with Differential Equations.

[36] Miller J.C. (2017). Mathematical models of SIR disease spread with combined non-sexual and sexual transmission routes. Infect. Dis. Model..

[37] Gao S., Teng Z., Nieto J.J., Torres A. (2007). Analysis of an SIR Epidemic Model with Pulse Vaccination and Distributed Time Delay. J. Biomed. Biotechnol..

[38] Croccolo F., Roman H.E. (2020). Spreading of infections on random graphs: A percolation-type model for COVID-19. Chaos Solitons Fractals.

[39] Yang W., Zhang D., Peng L., Zhuge C., Hong L. (2020). Rational evaluation of various epidemic models based on the COVID-19 data of China 2020. arXiv.

[40] Hohenberg P.C., Halperin B.I. (1997). Theory of dynamic critical phenomena. Rev. Mod. Phys..

